# New Light on Prions: Putative Role of PrP^c^ in Pathophysiology of Mood Disorders

**DOI:** 10.3390/ijms25052967

**Published:** 2024-03-04

**Authors:** Adrian Andrzej Chrobak, Patrycja Pańczyszyn-Trzewik, Patrycja Król, Magdalena Pawelec-Bąk, Dominika Dudek, Marcin Siwek

**Affiliations:** 1 Department of Adult Psychiatry, Jagiellonian University Medical College, Kopernika 21A, 31-501 Kraków, Poland; adrian.chrobak@uj.edu.pl (A.A.C.); patrycja.krol@uj.edu.pl (P.K.); dominika.dudek@uj.edu.pl (D.D.); 2 Department of Human Physiology, Institute of Medical Sciences, Medical College of Rzeszow University, Kopisto 2a, 35-959 Rzeszow, Poland; ppanczyszyn@ur.edu.pl; 3 Department of Affective Disorders, Jagiellonian University Medical College, Kopernika 21A, 31-501 Kraków, Poland; magdalena.pawelec@uj.edu.pl

**Keywords:** PrP^Sc^, psychiatry, mood regulation, cognitive function, sleep and circadian rhythms, biomarker

## Abstract

Mood disorders are highly prevalent and heterogenous mental illnesses with devastating rates of mortality and treatment resistance. The molecular basis of those conditions involves complex interplay between genetic and environmental factors. Currently, there are no objective procedures for diagnosis, prognosis and personalization of patients’ treatment. There is an urgent need to search for novel molecular targets for biomarkers in mood disorders. Cellular prion protein (PrP^c^) is infamous for its potential to convert its insoluble form, leading to neurodegeneration in Creutzfeldt-Jacob disease. Meanwhile, in its physiological state, PrP^c^ presents neuroprotective features and regulates neurotransmission and synaptic plasticity. The aim of this study is to integrate the available knowledge about molecular mechanisms underlying the impact of PrP^c^ on the pathophysiology of mood disorders. Our review indicates an important role of this protein in regulation of cognitive functions, emotions, sleep and biological rhythms, and its deficiency results in depressive-like behavior and cognitive impairment. PrP^c^ plays a neuroprotective role against excitotoxicity, oxidative stress and inflammation, the main pathophysiological events in the course of mood disorders. Research indicates that PrP^c^ may be a promising biomarker of cognitive decline. There is an urgent need of human studies to elucidate its potential utility in clinical practice.

## 1. Introduction

Mood disorders are mental illnesses in which marked dysregulation of emotional states is the main clinical feature. The Diagnostic and Statistical Manual of Mental Disorders, Fifth Edition (DSM-5) classifies those conditions as bipolar and depressive disorders. They are characterized by the presence of depressive episodes, that manifests as persistently depressed mood and/or loss of interest, and are distinguished from each other by the presence of hypomanic/manic episodes, in which abnormally elevated, expansive or irritable mood is a dominant features [1,2]. Mood disorders are a global public health problem because of their devastating rates of prevalence, mortality, morbidity and treatment resistance. Depressive and bipolar disorders affects 4.4% and 2.4% of the global population, respectively [3,4], and these disorders are one of the leading causes of disability worldwide [5]. Despite the significant progress in the area of psychopharmacotherapy, approximately one-third of major depressive disorder patients and one-fourth of individuals with bipolar disorder present treatment resistance [6,7]. This may be related to the significant heterogeneity of those conditions and their complex pathophysiology. The molecular basis of mood disorders involves interplay between genetic, epigenetic and environmental factors, resulting in disrupted neurotransmission, oxidative stress and neuroinflammation. Despite the years of research devoted to the biological underpinnings of mood disorders, results did not lead to the identification of objective diagnostic procedures which could aid clinicians in the diagnosis, prognosis and personalization of patients’ treatment [8]. There is an urgent need to search for objective biomarkers for early diagnosis and more focused psychopharmacotherapy.

Cellular prion protein (PrP^c^) is a highly conserved cell-surface glycoprotein that is synthesized by most vertebrates at every phase of their development and in every tissue, especially in the central nervous system (CNS) [9]. PrP^c^ is infamous for its potential to convert into its scrapie conformation (PrP^Sc^) in an infectious-like, self-perpetuating mechanism that leads to rapidly progressive neurodegeneration observed in Creutzfeldt–Jacob disease (CJD). Meanwhile, the role of PrP^c^ in the physiological state remain elusive, and as research on it grows, the number of its possible functions increases. So far, it has been identified that PrP^c^ is responsible for, i.e., transmembrane signaling, binding of copper ions, adhesion of cells to the extracellular matrix, calcium homeostasis, maintenance and formation of synapses and protection against oxidative stress and apoptosis [10]. Through many interacting partners, this protein modulates neurotransmission, neuronal excitability and synaptic plasticity, thus influencing cognitive processes and regulation of behavior. Due to its neuroprotective activity and its involvement in numerous neuronal processes, a growing number of studies evaluates its role in the pathophysiology of CNS disorders such as Alzheimer’s disease, Parkinson’s disease, Huntington’s disease and amyotrophic lateral sclerosis [11]. Moreover, it has been shown that PrP^c^ may be a promising biomarker of multiple sclerosis, traumatic brain injury and concussion as well as an indicator of cognitive impairment in Parkinson disease and HIV-infected patients [12,13,14,15]. Surprisingly little research has paid attention to the role of PrP^c^ in mental disorders and their symptoms [16]. 

In this article, we aimed to integrate the available knowledge about molecular mechanisms in which PrP^c^ may have an impact on pathophysiology of mood disorders. We will discuss the role of PrP^c^ in the CNS, starting with its functions in chemical neurotransmission, moving through its impact on behavior and ending with a review of the human studies, emphasizing its promising role as a biomarker in neuropsychiatric research. A literature review was performed in November and December 2023 by analyzing eligible studies from databases (PubMed/MEDLINE, Google Scholar and Cochrane Library) using the following search terms: PrP^c^ AND one of the following keywords: depress*. bipolar*, cognitive funct*, sleep, behavior, biological rhythms, glutamate, serotonin, dopamine, acetylcholine, purine. No language or date filters were applied.

## 2. The Function of the Prion Protein (PrP^c^) in Chemical Neurotransmission

Prion diseases cause behavioral and neuropsychiatric symptoms that are characterized by rapid progression [17,18]. Hence, there is a critical need to explore and define the molecular mechanisms linked to PrP^c^ function in the pathophysiology of mood disorders. The effect of PrP^c^ on neurotransmitter regulation, including neurotransmitter synthesis, catabolism and release, appears to be the most important factor in the development of depressive symptoms. Several studies have shown that PrP^c^ is involved in synaptic transmission [19,20,21]. Steinert (2015) demonstrated that the presynaptic expression of a wild-type mouse prion protein at glutamatergic synapse led to an enhanced release of neurotransmitter molecules [20]. Similarly, qRT-PCR analysis indicated that PrP^c^ is committed to synaptic transmission by regulating Syngr3, Stx1b, Rab1a, Rab3a, Rab7a and Rab11b protein expression involved in synaptic vesicle exocytosis [19]. The synaptoplasmic localization of PrP^c^ and binding with synapsin 1 and synaptophysin are probably responsible for its action in neurotransmission [21]. Endogenous PrP^c^ function in synaptic signaling, including the regulation of synaptic density, may be directly related to the molecular background of mood disorders. Especially a lower synaptic density has been linked with the progression of depressive episodes [22].

Other reported PrP^c^ signaling pathways that are important in the regulation of chemical neurotransmission include phosphoinositide 3 kinase (PI3K), Akt protein kinase A (PKA), MAP kinase ERK1/2, mammalian target of rapamycin (mTOR) and calcium signaling—molecular mechanisms underlying the pathophysiology and treatment of mood disorders [23,24,25].

Notably, the functional interactions between PrP^c^ and neurotransmitter receptors implicated in depression have been investigated [26]. 

### 2.1. Monoamine Neurotransmitters

Currently, first-line pharmacotherapies for depression are based on monoamine neurotransmitters [27,28]. Thus, the fundamental questions are as follows: can PrP^c^ be a novel molecular target for the regulation of monoaminergic systems? In addition, can this intracellular pathway be a new mechanism relevant to antidepressant action? Beckman et al. (2015) have demonstrated the role of PrP^c^ in monoaminergic synapses [29]. Western blotting showed increased levels of dopamine, tyrosine hydroxylase and 5-HT_5A_ serotonin receptors in the cerebral cortex of knock-out mice (PrP^c^-null; PrP^−/−^). In brain tissue samples from PrP^c^-null mice, a significantly larger cAMP-dependent potentiation of neurotransmitters was observed when treated with low serotonin concentrations (1 nM). Interestingly, there were no significant differences in the cAMP response induced by norepinephrine or higher serotonin concentrations. In the context of the dopaminergic system, overlay assays of cerebrocortical protein extracts from PrP^−/−^ mice were positive for dopamine receptor D_1_ (but not D_4_) [29]. Dopamine (100 μM) reduced the levels of the unglycosylated form of PrP^c^ and mTOR. It also promotes autophagy (increased p62/SQSTM1 levels) [30]. The co-localization of dopaminergic neurons in the striatum of mice has been previously reported [31]. Simultaneously, the knock-out of PrP^c^ leads to the downregulation of dopamine D_1_ receptors and reduces dopamine levels in the prefrontal cortex of mice [31]. In turn, the synthetic stimulant—methamphetamine induces the accumulation of PrP^c^ in dopaminergic neurons (PC12 cell line) [32]. These results suggest that PrP^c^ functions in monoaminergic synapses may be linked to binding to both serotonergic and dopaminergic receptors. Moreover, this regulation depends on the monoaminergic system, receptor profile and intracellular concentrations of selective monoamines. 

### 2.2. Glutamatergic Neurotransmission

Dysfunction of glutamatergic neurotransmission is well established in depression [33]. Targeting the regulation of glutamate and glutamatergic-related pathways may contribute to the development of novel antidepressants (such as esketamine, approved by the FDA for the treatment of treatment-resistant depression) [34,35,36]. The interaction between PrP^c^ and glutamate receptors represents a new direction in neuropsychiatric research. 

PrP^c^ generally regulates N-methyl-D-aspartate receptors (NMDAR) function via S-nitrosylation, a copper-dependent reaction [37]. Huang et al. (2021) showed that the copper ion interaction of PrP^c^ is critical for affecting NMDA activity [38]. In PrP^c^-null mice, copper ions enhance the speed of NMDAR desensitization [39]. Notably, propagation of scrapie isoform of prion protein (PrP^Sc^) may induce a neurotoxic signal mediated by NMDAR [40]. This structural change in prion proteins is associated with cognitive impairment [41]. Fang et al. demonstrated that PrP^Sc^-related synaptotoxicity requires activation of NMDAR. Moreover, memantine (an NMDA receptor antagonist) prevents these effects [40]. According to the molecular background, the regulation of Ca^2+^ influx is essential for PrP^Sc^-induced NMDAR activation. An in vitro study showed that L-type calcium channels (LTCCs) and NMDAR activity modulate PrP^Sc^ formation (accumulation) via the ERK signaling pathway [42].

The neuroprotective effects of PrP^c^ represent a second mechanism involved in cognitive function [9]. PrP^c^ may prevent NMDAR hyperactivity and protect cells against glutamate excitotoxicity. Upregulation of NMDAR-containing GluN2D subunit was observed in the hippocampal neurons of PrP^c^-null mice [43]. MK801 (a highly potent, selective and non-competitive NMDA receptor antagonist) protects against increased cell death in transgenic mice subjected to kainate excitotoxicity [44]. 

Further research should focus on determining the role of PrP^c^ in regulating NMDAR activity and defining specific cell signaling pathways or post-translational modifications associated with neurotoxic and neuroprotective effects. This finding is significant because neurotoxicity leads to the progression of depressive disorders. In turn, mediating the neuroprotective pathway provides an opportunity to develop new pharmacological strategies.

PrP^c^ can also control the activity of α-amino-3-hydroxy-5-methyl-4-isoxazole propionic acid receptors (AMPARs) [45]. Tg mice expressing genetically modified PrP (CJD178 and FFI mutations) showed alterations in the structure and function of excitatory synapses through the trafficking of the GluA2-AMPAR subunit [46]. Furthermore, a significantly increased phosphorylation level of S845 GluA1 AMPAR subunit (pGluA1-S845) was observed in the hippocampus of prion-infected mice [47]. The study conducted by De Mario et al. (2017) suggested that PrP^c^ induced the downregulation of AMPARs, underlining its neuroprotective function [48]. CNQX (a competitive AMPA receptor antagonist) induces protective effects by preventing the PrP^Sc^-induced retraction of dendritic spines [39]. Similar to NMDA, the Ca^2+^ flux is an important mechanism involved in the regulation of AMPA activity. In the same study, De Mario et al. (2017) showed that PrP^c^ takes part in Ca^2+^ homeostasis regulation and contributes to the modified synaptic activity of ionotropic glutamate receptors [48].

Importantly, PrP^c^ participates in the postsynaptic regulation and interaction of group I metabotropic glutamate receptors (mGluRs) [49,50,51]. Recent studies have revealed that PrP^c^ accumulation is related to abnormal mGluR1 signaling [49,52]. These relationships have also been demonstrated in the pathophysiology of Alzheimer’s disease (see [53,54] for review). Goniotaki et al. highlighted the role of pharmacological inhibition of mGluR1 and mGluR5 in reducing the dysfunction associated with prion diseases. For example, the results indicated that MPEP (2-Methyl-6-(phenylethynyl)pyridine; selective mGluR5 antagonist) prevents neurodegeneration in organotypic slice cultures [49]. Further studies have demonstrated that the N-terminal region of PrP^c^ is critical for forming a complex with mGluR1. In addition, PrP^c^ modulates the mGluR1 function to prevent abnormal Ca^2+^ signaling [49]. Moreover, reduced mGluR5 dimers were observed in the hippocampus of male wild-type mice and the occipital cortex of patients with sporadic Creutzfeldt–Jakob disease. This evidence also suggests that prions trigger an early Arc response that consistently disrupts glutamatergic synaptic homeostasis [47].

### 2.3. Cholinergic and Purinergic Neurotransmission

PrP^c^ function is linked to regulation of M1 muscarinic acetylcholine receptor (M1 mAChR) activation. Dwomoh et al. showed that the administration of VU0486846 (an M1-positive allosteric modulator) reduced neuroinflammation and mitochondrial markers in the hippocampus of prion-infected mice [55]. M1 muscarinic allosteric modulators can slow prion-induced neurodegeneration [56,57]. Additionally, the regulation of the nicotinic acetylcholine receptor (α7nAchR) by prion PrP^c^ may be an alternative mechanism underlying neuroprotection [58,59]. 

Finally, PrP^c^ selectively binds to the purinergic receptor P2X4. This interaction has been proposed as a mechanism linked to proteostasis dysfunction and the progression of neurodegeneration [60].

In summary, the physiological function of PrP^c^ and its effects on the central nervous system are related to the regulation of neurotransmitters at both pre-and postsynaptic levels [61]. Interestingly, PrP^c^ modulates all the chemical neurotransmitters linked to specific neuropsychiatric conditions. The molecular mechanisms underlying interactions of PrP^c^ with selected neurotransmitter receptors are not fully understood. Among the defined molecular pathways, monoaminergic control of intracellular cAMP [29] cooper-dependent modulation of NMDA receptor function [38] and regulation of post-translational modifications in the synaptic complex of AMPA, NMDA and mGluR receptor activity [47,49] seem to be the most critical. Increasing evidence suggests that initiation of prion protein misfolding (accumulation of PrP^Sc^) leads to oxidative stress and neuroinflammation [62,63]. Assessing the role of the nuclear factor erythroid2-related factor-2 (Nrf2) and p62-Keap1-NRF2-ARE pathways in the regulation of PrP^c^ expression is also an essential aspect in the context of the pathogenesis of depressive, cognitive or schizoaffective symptoms [25]. These mechanisms may indirectly affect the neurotransmitter systems. The role of PrP^c^ in neurotransmitter modulation and its importance as a novel therapeutic target in antidepressant drug action require further detailed molecular and biochemical investigations. The functions of the prion protein (PrP^c^) in chemical neurotransmission have been summarized in Table 1.

## 3. The Function of the Prion Protein (PrP^c^) in Depressive-like Behavior Regulation, Cognitive Functioning, Sleep and Circadian Rhythms

Most of our knowledge about the role of PrP^c^ stems from experiments with transgenic animal models with knock-out of the PrP^c^ gene. An increasing body of research suggests a potential involvement of this protein in anxiety, nesting, aggression, depression and cognitive functions, including memory and associative learning. Animal studies have further demonstrated that the absence of PrP^c^ leads to disturbances in olfactory function and behavior [67] and alterations of circadian rhythms and changes in sleeping patterns. In this section, we will discuss in detail the role of PrP^c^ in the regulation of depressive-like behavior and its role in core areas of functioning related to the symptomatology of mood disorders such as cognition, sleep and circadian rhythms [68]. 

### 3.1. Depressive-like Behavior Regulation

Depressive-like behaviors are common in prion diseases [18], and among widespread neurodegeneration, a reduction in both monoaminergic cells and associated markers has been detected in the brains of individuals affected by these illnesses [69,70]. Transgenic mice with knock-out of the PrP^c^ gene exhibited depressive-like behavior that was reflected in extended immobility time in both the forced swimming test (FST) and tail suspension test (TST) [71]. The administration of the tricyclic antidepressant imipramine and the NMDAR antagonist MK-801 effectively reversed the depressive-like behavior observed in the knock-out mice during the TST. These findings emphasize the significant role of PrP^c^ in pathophysiology of a depressive-like state in mice, supporting the notion that alterations of this protein may contribute to depressive-like disorders. Furthermore, the results suggest that PrP^c^ could be considered as a potential target for drug interventions in the treatment of depressive disorders [71].

It has been shown that PrP^c^ modulates behavioral reactions induced by innate fear. Lobão-Soares at al. utilized wild-type (WT), PrP^c^ knockout (*Prnp*^−/−^) and PrP^c^ overexpressing Tg-20 mice in a prey versus predator paradigm, also assessing their behavioral performance in olfactory discrimination tasks. When exposed to coral snakes, both *Prnp*^−/−^ and Tg-20 mice demonstrated a significant decrease in the frequency and duration of defensive attention and risk assessment compared to WT mice. Tg-20 mice exhibited a lower frequency of escape responses, increased exploratory behavior and heightened interaction with the snake, suggesting a notable fearlessness linked to PrP^c^ overexpression. Notably, *Prnp*^−/−^ mice displayed a specific decrease in attentional defensive response (reduced frequency of defensive alertness) in the presence of coral snakes. Additionally, Tg-20 mice displayed increased exploration of novel environments and odors. These findings propose that PrP^c^ overexpression induces hyperactivity, fearlessness and an increased preference for visual, tactile and olfactory stimuli-related novelty. In summary, study suggest that PrP^c^ plays a vital role in regulating innate fear and exploration-driven by novelty [72].

### 3.2. Cognitive Functioning

The findings suggest that PrP^c^ is involved in maintaining cognitive functioning, with its absence potentially contributing to age-related declines in these functions and diminished resilience to neurological challenges [73]. PrP^c^-null mice displayed deficiencies in spatial learning and memory consolidation that rely on the hippocampus. The studies unveiled that mPrP^−/−^ mice exhibited a significant decline in both short- and long-term plasticity in dentate gyrus (DG) neurons located in the septal pole of the dorsal hippocampus. These shortcomings were rectified in transgenic mPrP^−/−^ mice, where PrP^c^ was introduced into neurons under the regulation of the neuron-specific enolase (NSE) promoter. This indicates that the deficits were a consequence of the PrP^c^ elimination in neurons [74]. The alterations observed were reversed when PrP^c^ was reintroduced [75]. 

On the other hand, over-expression of PrP^c^ induces hyperactivity and an elevated preference for visual, tactile and olfactory stimuli linked to novelty. Moreover, this reaction is influenced by the modulation of pathways associated with programmed cell death. Tg-20 mice, identified by a five-fold increase in PrP^c^ expression compared to wild-type mice, exhibited resilience to spatial learning and memory impairments induced by Aβ1–40. This resilience was evident in the form of reduced escape latencies to locate the platform and an increased duration spent in the correct quadrant during both the training and probe test sessions of the water maze task. The protective effect against cognitive impairments induced by Aβ1–40 in Tg-20 mice correlated with a significant decrease in the hippocampal expression of the activated caspase-3 protein and a reduction in the Bax/Bcl-2 ratio. Additionally, there was a mitigation of hippocampal cell damage, as determined through MTT and propidium iodide incorporation assays [76]. It is noteworthy that a five-fold surge in PrP^c^ expression, compared to wild-type mice, leads to heightened resilience against age-related cognitive decline [77].

The cerebellum is a preferential target of prions in Creutzfeldt–Jakob disease [78]. A growing number of studies indicate a significant role of this structure not only in motor coordination but also in regulation of emotions and cognition, particularly in executive functions [79], visuospatial functions [80], language [81] and memory [82] Moreover, it has been shown that the cerebellum may be related to the pathophysiology of bipolar disorder and schizophrenia, and the patients with those conditions present motor abnormalities related to the dysfunction of this structure [83,84,85,86,87,88,89,90]. PrP^c^ may play a role in the pathophysiology of mood disorders through its significant role in long-term depression (LTD), a critical cellular mechanism responsible for synaptic plasticity and neuroprotection against excitotoxicity in the cerebellum [91,92] Transgenic mice with knock-out of PrP^c^ gene (Ngsk *Prnp*^−/−^) presented impaired LTD in excitatory synaptic transmission at connections between parallel fibers and Purkinje cells. Additionally, GABA-A-mediated inhibitory postsynaptic currents recorded from Purkinje cells were attenuated in Ngsk *Prnp*^−/−^ mice. Those changes resulted in alterations in eyeblink conditioning, a motor learning paradigm related to cerebellar plasticity, indicating a significant role of PrP^c^ in cognitive deficits associated with this structure [93].

Given the diverse functions of PrP^c^, there are numerous potential molecular mechanisms through which this protein influences cognitive processes. PrP^c^ is situated in the synaptic area, where it has the potential to interact with other proteins within this structure, thereby playing a significant role in synaptic plasticity [94]. In an in vitro investigation, recombinant PrP has been shown to rapidly stimulate the development of both axons and dendrites, along with promoting the generation of new synapses. An extended overnight exposure to recombinant PrP from Syrian hamster or mouse, folded into a conformation rich in α-helices resembling that of PrP^c^, resulted in 1.9-fold elevation in neurons displaying a differentiated axon, a 13.5-fold rise in neurons with differentiated dendrites, a 5-fold rise in axon length and the establishment of extensive neuronal circuitry. Additionally, the formation of synaptic-like contacts witnessed a 4.6-fold elevation after a 7-day exposure to recombinant PrP [95]. The deletion of PrP^c^ led to impaired long-term potentiation, coupled with a reduction in fast GABA-A receptor-dependent inhibition [96,97]. Possibly, alterations in long-term potentiation may be linked to the capability of PrP^c^ to physically interact with glutamate receptors. Experimental evidence indicates that PrP^c^ can co-immunoprecipitate with the GluN2D subunit of the NMDA receptor, suggesting its potential for direct modulation [98]. Transgenic mice with knock-out of the PrP^c^ gene exhibited heightened NMDA-induced currents, and this effect was reversed upon the overexpression of PrP^c^ [43]. PrP^c^ also influences kainate receptors [44] and metabotropic glutamate receptors [59], exerting a suppressive effect on neuronal excitability through various mechanisms. The regulation of glutamate signaling within synapses is crucial not only for synaptic plasticity but also because abnormal calcium (Ca^2+^) currents, induced by the inappropriate activation of the NMDA receptor, can lead to excitotoxicity [99] and as a result to neurodegeneration.

The performance of older mice, particularly those with knock-out of the PrP^c^ gene, showed impairment in tests evaluating different subdomains of motor function. These tests encompassed assessments of locomotion, strength, balance and coordination [73,77,100,101,102,103,104]. Mice with knocked-out PrP^c^ were found to be more susceptible to age-related declines in both memory [102] and motor processes [105].

### 3.3. Sleep and Circadian Rhythms

Sleep disturbances are observed in 60–90% of patients with major depressive disorder and in more than 90% of individuals with bipolar depression [106]. The PrP gene appears to be involved in regulating sleep patterns and circadian rhythms that are strictly associated with the symptomatology of affective disorders [107,108,109,110]. Certain findings indicate that neuronal PrP^c^ plays a role in sleep homeostasis and sleep continuity, whereas non-neuronal PrP^c^ does not appear to be involved. These findings further propose that the hormonal regulation of the hypothalamic–pituitary–adrenal (HPA) axis, dependent on neuronal PrP^c^, may contribute to the maintenance of sleep homeostasis. Following sleep deprivation, mice with knock-out of the PrP^c^ gene exhibited more pronounced sleep fragmentation and longer latency to enter rapid eye movement (REM) and non-REM sleep (NREM) compared to wild-type mice. During sleep recovery, mice null for PrP^c^ showed a reduction in the amount of NREM sleep and slow-wave activity. Additionally, after sleep deprivation, the patterns of corticosterone (CORT) and adrenocorticotropic hormone (ACTH) levels differed between wild-type mice and those with knock-out of the PrP^c^ gene. The profile of ACTH/CORT indicated a pivotal role of PrP^c^ in regulating the negative feedback loop of glucocorticoids on ACTH and corticotropin-releasing factor (CRF) [111].

Laminin (LN) is recognized for its ability to enhance neurite outgrowth and facilitate memory consolidation, whereas amyloid-beta oligomers (Aβo) induce synaptic dysfunction. In both these pathways, mGluR1 serves as a co-receptor. The participation of the PrP^c^/mGluR1 complex in these divergent functions implies that this interaction is a pivotal element in regulating synaptic activity. An inquiry was conducted to explore the impact of sleep deprivation on the expression of PrP^c^ and its associated elements, including laminin, Aβo and mGluR1. This multicomplex has the potential to disrupt neuronal plasticity, and the study aimed to understand how sleep deprivation influences the levels of these key components. Sleep deprivation resulted in a decrease in the levels of PrP^c^ and mGluR1, with a more pronounced impact observed during the active state. Sleep deprivation also caused accumulation of Aβ peptides in rest period, while laminin levels were not affected. An in vitro binding assay showed that Aβ can compete with LN for PrP^c^ binding. The results suggest that sleep deprivation has a regulatory influence on the levels of PrP^c^ and Aβ peptides. According to the assays, these changes induced by this process may have adverse effects on the interaction between laminin and PrP^c^, a relationship known for its involvement in neuronal plasticity [112].

In the genetic prion disease known as fatal familial insomnia (FFI), a prominent clinical feature is a profoundly disrupted sleep. This is characterized by insomnia resistant to anxiolytics, dysfunction of circadian rhythms, sleep fragmentation and altered arousal. Polysomnographic studies in FFI-affected patients reveal a reduction in total sleep time, decreased REM (rapid eye movement) sleep, and the loss of REM atonia, emphasizing the severity of sleep disturbances associated with this condition [113]. In the clinicopathology of fatal familial insomnia (FFI), central sleep apnea and a reduction in slow-wave sleep are additional common features. These observations contribute to the overall characterization of the severe and complex sleep disturbances experienced by individuals affected by FFI [114]. During clinical evaluation, nearly 90% of individuals with sporadic CJD (spCJD) reported sleep dysfunction. This high prevalence underscores the significance of sleep disturbance as a prominent and widespread symptom in spCJD patients, surpassing the occurrence of other diagnostic criteria for CJD [115]. In a separate study, sleep disturbances were identified as prevalent clinical complaints among all familial CJD patients who were examined. This suggests that sleep-related issues are a common and noteworthy aspect of the clinical presentation in both sporadic and familial forms of CJD [116]. Several animal models of prion disease exhibit disrupted sleep patterns. For instance, rats inoculated with various prion strains demonstrate significant decreases in slow-wave sleep [117]. Rhesus monkeys infected with the human prion disease kuru exhibit a complete loss of REM sleep and disrupted sleep stage cycling [118]. Additionally, mice inoculated with the murine prion disease RML display alterations in rest period activity, with changes evident from very early in the incubation period [119]. These observations highlight the impact of prion diseases on sleep patterns across different animal models. Reports indicate that patients with Gerstmann–Sträussler–Scheinker disease (a genetic prion disease primarily characterized by ataxia and pyramidal dysfunction) do not demonstrate sleep alterations [120,121]. Both clinical observations and experimental models of prion disease provide evidence of clear circadian dysfunction. This implies a potential role for PrP^c^ in the synthesis or regulation of melatonin release in health [122]. Table 2 summarizes studies evaluating the role of PrP^c^ in regulation of depressive-like behavior, cognitive functioning, sleep and circadian rhythms.

## 4. Human Studies

Weis et al. (2008) performed the first neuropathological evaluation of the PrP^c^ expression in the brain of the patients with mood disorders. Authors measured immunoreactivity of this protein in the anterior cingulate gyrus derived from the 15 patients with bipolar disorder, 15 patients with major depressive disorder, 15 patients with schizophrenia and 15 controls. It has been shown that PrP^c^-positive glial cells in white matter were reduced in the three disorders examined, while no significant changes were observed in gray matter. The patomechanism and relevance of the abovementioned findings are still unknown [124]. Studies indicate that PrP^c^ may play a role as a neuroprotective factor. It has been shown that through interactions with GluN2D and GluR6/7 receptors, this protein blocks the neurotoxicity induced by NMDA and Kainate. *Prnp* knock-out mice present enhanced susceptibility to excitotoxic insults that can be rescued by overexpression of PrP^c^ from exogenous *Prnp* cDNA [43,125]. This protein shows a protective role against oxidative stress. It has been shown that that activity of one of the major enzymes detoxifying reactive oxygen species, Cu/Zn superoxide dismutase (SOD), decreases dramatically in PrP^c^ knock-out mice and increases with its overexpression [126]. This protein may protect cells from inflammation, and its absence has been shown to exacerbate and prolong neuroinflammation [127]. PrP^c^ has been shown to regulate innate immune system functions. Through the interaction with NMDA receptors in macrophages, this protein may block inflammatory cytokine mRNA expression, leading to broad anti-inflammatory activity [128]. This observation is particularly important, as the activation of innate immune mechanisms, especially proinflammatory cytokines Il-1, Il6 and tumor necrosis factor alpha (TNF-α), may contribute to the initiation and progression of mood disorders through their impact on the inter alia sympathetic system, hypothalamo–pituitary–adrenal axis and serotonergic pathways, leading to increased synthesis of catecholamines, glucocorticosteroids and synthesis of neurotoxic tryptophan metabolites [129]. There is also evidence suggesting that PrP^c^ also contributes to neuroprotection by regulating intracellular signaling cascades that mediate cellular survival, as its overexpression has been shown to provide protection to cell lines from undergoing apoptosis [126]. Despite differences in pathophysiology between schizophrenia, bipolar disorder and major depressive disorder, abovmentioned processes, namely excitotoxicity, oxidative stress and neuroinflammation, are core factors associated with neurodegeneration observed in those conditions [130,131]. PrP^c^ immunoreactive neurons observed in the anterior cingulate gyrus of evaluated clinical groups may represent the population of cells that escaped the abovementioned processes due to PrP^c^ upregulation. This protective mechanism may be hampered in white matter of the patients with major depressive disorder, schizophrenia and bipolar disorder [124].

While there is a growing amount of research evaluating the role of PrP^c^ in CSN functions, surprisingly little research has assessed its role as a biomarker in humans. One of the most important studies has been conducted by Breitling et al., in which authors examined the association between serum PrP^c^ and cognitive performance [132]. This was a cross-sectional study performed on 1322 German elderly individuals of age 65+. For the assessment of the cognitive functions, the authors used a brief telephone interview (COGTEL) consisting of six components evaluating the following cognitive functions: prospective memory, verbal short-/long-term memory, verbal fluency, working memory, inductive reasoning. In the study, an overall inverse and pronouncedly non-linear association of serum PrP^c^ with the score of cognitive functioning was found: the higher concentrations of this protein, the lower the overall cognitive performance. This study provided the first evidence that readily accessible serum PrP^c^ may constitute a biomarker of cognitive functioning [132]. A recent study of Leng et al. proposed a novel method for analyzing PrP^c^ in blood by assessing its concentration in plasma exosomes [13]. Those structures are nano-sized extracellular vesicles that are released by most of the cell types to the extracellular matrix and may be found in blood [133]. It has been shown that PrP^c^ is highly expressed in exosomes, and their concentration may reflect the intracellular environment of the originating cells [134,135]. Leng et al. have shown that plasma exosomal prion protein levels differentiate Parkinson’s disease patients from healthy controls. Moreover, its concentration correlates with the severity of cognitive impairment, particularly in visuospatial, memory and calculation abilities domains [13]. In Parkinson’s disease, PrP^c^ interacts with a-synuclein, a neuronal protein responsible for synaptic vesicle trafficking, which aggregates into Lewy bodies that are associated with neurodegeneration in this condition. PrP^c^ promotes uptake of fibrillar forms of this protein and is responsible for alfa-synuclein-dependent impairment of hippocampal LTP, indicating its important role in pathology of cognitive impairment in Parkinson’s disease [136,137]. A study suggests that plasma exosomal concentration of PrP^c^ may serve as a biomarker of cognitive decline in this disorder [13]. Cognitive impairments are prevalent and debilitating symptoms in affective disorders [138]. Further studies should evaluate whether serum PrP^c^ concentration may be used as a biomarker of the severity of those symptoms among patients diagnosed with mood disorders.

A growing number of studies point out the significant role of NMDAR in the pathophysiology and the treatment of major depressive disorder. It has been shown that ketamine, an antagonist of this receptor, promotes rapid antidepressant and antisuicidal effect. A recent study suggests that PrP^c^ may play a mediating role in the molecular mechanism of this drug. It has been shown that depressive-like behavior caused by PrP^c^ depletion may be treated with the NMDAR antagonist [139]. PrP^c^ interacts with NMDA receptors in a copper-dependent manner. In its copper-loaded state, PrP^c^ binds to NMDA receptors, decreasing the co-agonistic effect of glycine for this receptor [140]. Additionally, copper directly interacts with NMDAR as an antagonist [131,141]. Thus, it is plausible to hypothesize that there is a synergistic interaction between ketamine, copper and PrP^c^–copper complexes leading to antidepressant effect [139].

## 5. Conclusions

Our review indicates an important role of PrP^c^ protein in the molecular mechanisms of neurotransmission and synaptic plasticity. This protein is involved in major processes responsible for the regulation of cognitive functions, emotions, sleep and biological rhythms, and its deficiency results in depressive-like behavior and cognitive impairment. This protein also plays an important neuroprotective role against excitotoxicity, oxidative stress and inflammation, the main pathophysiological events in the course of mood disorders. Research indicates that PrP^c^ protein may be a promising biomarker of cognitive decline in neuropsychiatric conditions. There is an urgent need of human studies to elucidate its potential utility in clinical practice.

## Figures and Tables

**Table 1:** Summary of studies on the role of PrP^c^ in chemical neurotransmission regulation.

Neurotransmitter System	Species/Strains	Samples	Methods	Results	References
Serotonergic system	PrP^c^ knock-out mice (Npu *Prnp*^−/−^) (8–12-week-old male)	Cerebral cortex	Western blot	↔ 5HT1A receptors ↑ 5HT5A receptors ↔ SERT ↑ TH ↔ TPH	[29]
		Cerebral cortex	HPLC coupled with HPLC-ED	↔ 5HT, 5-HIAA	
		Cerebral cortex; SB-699551-A (5HT5A receptor antagonist—100 nM)	Accumulation of cAMP	↑ cAMP production stimulated by 1 nm serotonin	
		Cerebral cortex	Immunohistochemistry	colocalization of PrP^c^ with 5HT5A receptors	
		Cerebral cortex	Overlay assay	binding of PrP^c^ to 5HT5A receptors and SERT	
	PrP^c^ knock-out mice (3-month-old male)	Behavioral tests	5-HT_1B_R antagonist SB224289 (100 nM) in the substantia nigra injection; behavioral outcome	↓ frequency and time spent in open arms (EPM) ↓ overall distance (EPM)	[64]
Dopaminergic system	PrP^c^ knock-out mice (Npu *Prnp*^−/−^) (8–12-week-old male)	Cerebral cortex	Western blot	↔ D1 receptors	[29]
		Cerebral cortex	HPLC coupled with HPLC-ED	↑ DA	
		Striatum	HPLC coupled with HPLC-ED	↔ DA, DOPAC	
		Cerebral cortex	Immunohistochemistry	colocalization of PrP^c^ with D1 receptors	
		Cerebral cortex	Overlay assay	binding of PrP^c^ to D1 receptors	
	PrP^c^ knock-out mice (*Prnp*^−/−^) (2–3-month-old female)	Olfactory bulbectomy (OB), striatum (STR), hippocampus (HIP) and prefrontal cortex (PFC)	Western blot	↓ TH (OB, PFC) ↔ TH (STR, HIP) ↓ DA (OB, PFC) ↔ DA (STR, HIP)	[31]
			Autoradiography	↓ D1 receptors (STR) ↔ D2 receptors, DAT (STR)	
Glutamatergic system NMDA receptor	PrP^c^ knock-out mice (*Prnp*^−/−^; on a C57BL6 background)	Hippocampal neurons; treated for 24 h with purified PrP^Sc^	Western blot	↓ GluN1	[40]
	PrP^c^ knock-out mice (The FVB *Prnp*^−/−^)	Hippocampus	Western blot	↓ S-nitrosylation of GluN1 ↓ S-nitrosylation of GluN2A	[65]
		Organotypic hippocampal cultures (OHC) by analyzing neuronal death in Cornus Ammonis 1 (CA1), Cornus Ammonis *3* (CA3) and dentate gyrus (DG) treated with 5 μM NMDA for 3 h or 10 μM NMDA for 10 min	Confocal microscope fluorescence	↑ neuronal cell death (CA1, CA3, DG regions—after 5 µM and 10 µM NMDA exposure)	
	Tg650 PrP^c^ knock-in mice (with human cellular prion protein) and Tga20 PrP^c^ knock-in mice (over-expressing murine cellular prion protein)	Hippocampal neurons for primary culture	Whole-cell voltage-clamp	↑ sensitivity to glycine of NMDA receptors (from Tg650 mouse neurons)	[39]
Glutamatergic system AMPA receptor	Tg(CJD-A66^+/−^) and Tg(FFI-26^+/−^) expressing PrP at ~2X	Hippocampus (co-immunoprecipitate)	Immunoprecipitation/ Western blot	co-immunoprecipitated with GluA2 but not with GluA1	[46]
	Tg(PG14-A3^+/−^) expressing transgenic PrP at ~1X	Cerebral cortex (co-immunoprecipitate)	Immunoprecipitation/ Western blot	co-immunoprecipitated with GluA2	
	Tg(PG14-A3^+/−^) expressing transgenic PrP at ~1X	Cerebellar granule neurons	LDH assay	more vulnerable to the toxicity of glutamate and AMPA	
	Tg650 PrP^c^ knock-in mice (with human cellular prion protein) and Tga20 PrP^c^ knock-in mice (over-expressing murine cellular prion protein)	Hippocampal neurons for primary culture	Whole-cell voltage-clamp	↑ steady-state AMPA current (Tg650)	[39]
	C57BL/6J mice (6–8 weeks old)	Hippocampus	Western blot	↔ GluA1 ↑ pGluA1-S845	[47]
Glutamatergic system metabotropic glutamate receptors (mGluRs)	C57BL/6J mice (6–8 weeks old)	Hippocampus	Western blot	↓ mGluR5 monomer ↓ mGluR5 dimer	[47]
			Real-time quantitative reverse transcription PCR	↔ *Grm5* mRNA	
	PrP^c^ knock-out mice (on a C57/Bl6J background)	Synaptoneurosome of acute mouse brain slices	Immunoprecipitation/ Western blot	co-immunoprecipitation (co-IP) between PrP^c^ with mGluR5 receptors	[66]
	PrP^c^ knock-out mice (*Prnp*^−/−^)	Brain lysates (total extracts; TEs)	Immunoprecipitation/ Western blot	PrP^c^ interacts with mGluR1 and mGluR5 receptors ↔ mGluR1, mGluR5 receptors	[50]
Cholinergic and purinergic systems	Knock-in mice expressing M1-PD receptors	Hippocampus	Western blot	↑ PrP^Sc^ ↑ PrP^tot^.	[56]
	Knock-in mice expressing M1-PD receptors	Cornus Ammonis 1 (CA1)*,* Cornus Ammonis 3 (CA3) and dentate gyrus (DG)	Antibody-based biosensor of receptor activation; following fear-conditioning training	↑ M1 mAChR	[57]
	Lister hooded rats	Cerebellum	Overlay assay	detection of P2X4R bound to PrP^c^ but not of P2X7	[60]

Abbreviations: ↑ increase; ↓ decrease; ↔ no change compared to the control group (wild-type mice); serotonin transporter (SERT); tyrosine hydroxylase (TH); tryptophan hydroxylase (TPH); total serotonin level (5HT); 5-hydroxyindoleacetic acid (5-HIAA, from serotonin); high-performance liquid chromatography (HPLC); high-performance liquid chromatography coupled with electrochemical detection (HPLC-ED); elevated plus-maze (EPM); total dopamine level (DA); 3,4-dihydroxyphenylacetic acid (DOPAC, from dopamine); dopamine transporter (DAT); misfolded prion protein (PrP^Sc^); total prion protein (PrP^tot^); lactate dehydrogenase (LDH); muscarinic acetylcholine receptor (mAChR).

**Table 2:** Summary of the studies evaluating the function of the prion protein (PrP^c^) in depressive-like behavior regulation, cognitive functioning, sleep and circadian rhythms.

Category	Species/Strains	Methods	Results	References
Olfactory behavior and physiology	Wild-type mice: C57BL/6J × 129/Sv Knock-out mice: Zürich I *Prnp*^−/−^; Nagasaki *Prnp*^−/−^; Edinburgh *Prnp*^−/−^; Prn knockout Transgenic mice: Tg20; NSE-PrP; MBP-PrP; Tg306; Tg33	Behavioral tests: cookie finding behavior test; Habituation–dishabituation test Odor delivery: 2 s odor puffs at least seven times Electrophysiology recordings: craniotomies for electrode insertion into the main olfactory bulb and the lateral olfactory tract Local field potential signal processing and analysis	The absence of PrP^c^ leads to disturbances in olfactory function and behavior	[67]
Depressive-like behavior regulation		Neuropathologic evaluation; cell counting	There is a possible pathophysiological overlap of abnormal protein aggregation in CJD and Parkinson’s disease.	[70]
	Wild-type and PrP-null mice (C57BL/6J) (10 weeks old, weighing 25–30 g)	Drugs and treatment: Imipramine and MK-801; (dissolved in phosphate-buffered saline; intraperitoneal; 30 min before tests; 10 mL/kg body weight) Behavioral tests: forced swimming test; tail suspension test; open-field test	Transgenic mice with knock-out of the PrP^c^ gene exhibited depressive-like behaviors	[71]
	PrP^c^ knock-out mice (*Prnp*^−/−^, descendants of Zrch I) (adult male, 3 months old, weighing 30–40 g) Wild-type mice (129/Sv × C57BL/6J) (*Prnp*^+/+^, male, 30–50 g) Tg-20 mice (deletion of the kanamycin gene from knock-out mice and insertion of four *Prnp* genes) (*Prnp*s)	Behavioral tests: exposing different mouse strains to coral snakes (15 min confrontation) Predatory and antipredatory behavioral recordings (15 min) Olfactory discrimination task (to discard potential olfactory discrimination deficits; cages with familiar and non-familiar odors; time recording)	PrP^c^ modulates behavioral reactions induced by innate fear	[72]
Cognitive functioning	mPrP^−/−^ mice (129/Ola background) mPrP^+/+^ mice (129/Ola background) mPrP^−/−^ mice in the mixed (129/Ola C57BL/10) background mPrP^−/−^ expressing a hamster PrP^c^ transgene mice (NSE-hPrP^c^/mPrP^−/−^) C57BL/10 mice (mPrP^+/+^ controls) (male and female ~5-month-old mice)	Behavioral tests: spatial version of the Barnes circular maze; non-spatial version of the Barnes circular maze Acute electrophysiological studies	PrP^c^-null mice displayed deficiencies in spatial learning and memory consolidation that rely on the hippocampus	[74]
	PrP^c^-deficient mouse line: Zrch *Prnp*^−/−^ and Ngsk *Prnp*^−/−^ Transgenic mouse line carrying the wild-type MoPrPA gene [Tg(MoPrP-A)B4053]	Samples: hippocampal slices Western blot Electrophysiological recordings	The mouse hippocampus exhibits an enhancement of excitatory synaptic transmission, mediated by the prion protein, dependent on the administered dose	[75]
	*Prnp*-null mice (*Prnp*^−/−^, descendants of Zrch I) Wild-type mice (*Prnp*^+/+^, 129/Sv × C57BL/6J) PrP^c^-overexpressing Tg-20 mice (4-month-old male, weighing 30–40 g)	Drug treatment: human Ab1–40 and the inverse peptide Ab40–1 (concentration 1 mg/mL; diluted in 0.1 mol/L phosphate-buffered saline (PBS); intracerebroventricular (i.c.v.) microinjections; 3 µL of PBS, Ab1–40 or Ab40–1; directly into the lateral ventricle; experiments: at least 14 days after administration) Behavioral tests: water maze task Samples: hippocampal slices Cell viability assays: MTT (3-(4,5-dimethylthiazol-2-yl)-2,5-diphenyltetrazolium bromide) cell viability assay; propidium iodide staining Western blot	Overexpression of cellular prion protein in mice acts as a safeguard against spatial learning and memory impairments	[76]
	*Prnp*-null mice (*Prnp*^−/−^, descendants of Zrch I) Wild-type mice (*Prnp*^+/+^, 129/Sv × C57BL/6J) PrP^c^-overexpressing Tg-20 mice (11-month-old male, weighing 30–40 g)	Drug treatment: PepSTI1230–245 and PepSTI1422–437 peptides (concentration 50 ng/µL; intracerebroventricular (i.c.v.) microinjections; 3 μL of PBS, PepSTI-1230–245 or PepSTI1422–437; directly into the lateral ventricle; the first juvenile presentation: 5 min after administration) Behavioral tests: open field; activity cages; elevated plus-maze; social recognition task; step-down inhibitory avoidance task Samples: blood (for the acetylcholinesterase (AChE) activity assay); hippocampus AChE activity assessment Immunohistochemistry: at the dorsal hippocampus	PrP^c^ plays a crucial role in the age-related behavioral deficits observed in mice	[77]
	Ngsk *Prnp*^−/−^ mice *Prnp*^+/−^ mice (F3 Ngsk × C57BL/6J) Ngsk *Prnp*^+/+^	Samples: cerebellum Electrophysiology: whole-cell voltage-clamp recordings from Purkinje cells; parallel fibers-evoked excitatory postsynaptic currents measurement, addition of bicuculline (10 μM) to the artificial cerebrospinal fluid; long-term depression induction; measurements of inhibitory postsynaptic currents Behavioral tests: eyeblink conditioning	Significant role of PrP^c^ in cognitive deficits associated with Purkinje cells	[93]
	Rats	Samples: hippocampus Protein production: full-length recombinant mouse PrP [MoPrP(23–230)] expressed from pET11a plasmid; recombinant full-length Syrian hamster PrP [SHaPrP(29–231)] and C-terminal region [SHaPrP(90–231)] produced from plasmid pNT3A Purification (MoPrP(23–230); SHaPrP(29–231) and SHaPrP(90–231); MoDpl(27–155); synthetic SHaPrP(23–98) peptide) Neuronal culture: protein and inhibitor treatment (RecPrP, recDpl, control proteins, kinase inhibitors) Immunofluorescence analysis	The introduction of recombinant prion protein prompts rapid polarization and the formation of synapses	
	PrP-null mice (*Prnp*^−/−^, 129/Sv × C57BL) (male, 12–16 weeks old) Wild-type mice (*Prnp*^+/+^, C57BL × 129/Sv) (male, 12–16 weeks old)	Samples: hippocampus Electrophysiological recordings (intracellular electrophysiological recordings on individual CA1 pyramidal cells)	The deletion of PrP^c^ led to impaired long-term potentiation	[96]
	PrP null mice (adult male) 129/Sv × C57BL/6J (control) mice (adult male)	Samples: hippocampus Electrophysiological recordings	The deletion of PrP^c^ led to impaired long-term potentiation	[97]
	PrP-null mice (PrP^−/−^, Zürich 1 strain) Wild-type mice (PrP^+/+^, 129 and FVB background)	Samples: hippocampus Neuronal primary culture and transfection: electroporation for introducing exogenous plasmids (cDNA or siRNA) into cells; enhanced YFP as a transfection marker Molecular biology: mouse *Prnp* cDNA cloned into pCMV-SPORT6 for PrP reconstitution experiments; siRNA experiments with retroviral short hairpin RNA (shRNAmir) constructs; NR2D RNAi experiments with specific shRNAmir constructs; RT-PCR to analyze NMDAR subunit and PrP expression Electrophysiological recordings Immunofluorescence microscopy Western blot ELISA assays and in vitro excitotoxicity assays (NMDA excitotoxicity assays) Lesion surgery and fluoro-jade staining (hippocampal injections of NMDA; staining to assess cell death) Spectral analysis of epileptiform discharges (Fourier-based analysis)	The prion protein mitigates excitotoxicity by suppressing NMDA receptor activity	[43]
	PrP^c^-deficient mice (*Prnp*^−/−^) 21 C57BL6J mice	Kainic acid injections (glutamate agonist KA; intraperitoneal (i.p.); 8 mg/kg b.w. every 30–60 min for up to 4 h; 0.1 M phosphate-buffered saline (PBS)) Seizure severity scoring (for 7 h after the first injection) Pharmacologic treatments of slice cultures (propidium iodide uptake after glutamate or KA treatment; use of the non-competitive NMDA receptor antagonist MK801) Histologic methods (antibodies used for immunostaining: a-fos, a-GluR1, a-GluR2/3, a-GluR4, a-GluR6-7, a-ERK1-2, a-p38, JNK, a-GFAP) Fluoro-jade B staining of dying neurons in brain sections Cell culture and small interfering RNA (siRNA) transfection Semi-quantitative RT-PCR of AMPA/KA receptors Real-time PCR Western blot	PrP^c^ influences kainate receptors	[44]
	PrP^c^-null mice (*Prnp*^−/−^, descendants of Zrch I) Wild-type mice (*Prnp*^+/+^, 129/Sv × C57BL/6J)	Samples: hippocampus Neuritogenesis assays Immunofluorescence PKC activity measurement PKA activity measurement ERKI/2 activity measurement Ca^2+^ signaling and data analysis Transfection of HEK 293 cells with mGluR1 and mGluR5 and reconstitution of PrP^c^-Ln γ1 peptide signaling Coimmunoprecipitation assay Colocalization with mGluRs	PrP^c^ influences metabotropic glutamate receptors	[59]
	PrP^c^-null mice (*Prnp*^−/−^, 129/Sv and C57BL/6J) Wild-type mice (*Prnp*^+/+^, 129/Sv and C57BL/6J) Surgically implanted male Wistar rats (3 and 9 months old)	Surgical procedure (rats) (bilateral implantation of a 30 g cannula, 1 mm above the CA1 area of dorsal hippocampus) Behavioral tests: Open field test; elevated plus-maze; inhibitory avoidance training	Transgenic mice with knock-out of the PrP^c^ gene were found to be more susceptible to age-related declines in memory	[102]
	Mice disrupted for the PrP^c^ gene (*Prnp*^−/−^, 129/Sv × C57BL/6J) Wild-type (control) mice (129/Sv × C57BL/6J) (male, 9 months old)	Samples: hippocampus Electrophysiological procedures: long-term potentiation assessment In situ hybridization and immunological detection	The heightened glutamatergic transmission in the hippocampus of *Prnp*^−/−^ mice is associated with increased plasticity and prolonged persistence of dentate long-term potentiation	[103]
	Wild-type mice (WT, FVB strain) PrP-KO mice (carrying an FVB genetic background (F10)) Tg46 mice (re-introduction of the PrP gene into the F10 background, PrP^c^ at amounts similar to the natural levels)	Behavioral tests: pole test; forced swimming test; cookie-finding test; intruder test; predatory aggression test; open field test	As individuals age, the lack of the prion protein leads to changes in neural processing that affect the ability to adapt to new situations.	[104]
	*Prnp*^−/−^ mice (Zurich strain, mixed 129/Sv and C57BL/6) FVB/*Prnp*^−/−^ mice (*Prnp*^−/−^ mice repeatedly crossed with wild-type FVB animals) Tg(GSS)22 mice (homozygous for the MoPrP-P101L transgene, FVB/*Prnp*^−/−^ background)	Samples: brain Histopathologic analysis Semi-automated lesion profiling Behavioral tests: rotarod test	Transgenic mice with knock-out of the PrP^c^ gene were found to be more susceptible to age-related declines in motor processes	[105]
Circadian rhythms and sleeping patterns	PrP-deficient mice (*Prnp*^−/−^) (129/Ola, 15.4 ± 0.4 weeks, 28.0 ± 0.7 g) Wild-type mice (*Prnp*^+/+^) (129/Ola, 17.2 ± 1.0 weeks, 31.3 ± 0.8 g)	12 h light–dark cycle with specific housing, environmental and light conditions Surgery (implantation of two epidural EEG electrodes, EMG electrodes and a thermistor to measure brain temperature) Sleep deprivation EEG analysis EMG analysis Sleep fragmentation assessment Behavioral tests: free choice exploration test; passive avoidance test; delayed and immediate alternation procedure in a T-maze	The absence of PrP^c^ leads to alterations of circadian rhythms and changes in sleeping patterns	[68]
	PrP^c^-null mice (*Prnp*^−/−^, 129/Ola) Wild-type mice (*Prnp*^+/+^, 129/Ola) Transgenic mice (Tg)	Measurement of the motor activity rhythm In situ hybridization Implantation with chronical electrodes (for EEG and EMG recordings and with a cortical thermistor)	The PrP gene appears to be involved in regulating sleep patterns and circadian rhythms	[123]
	mPrP^0/0^ mice (129/Ola) Wild-type controls: C57BL/10 (BL10 mPrP^+/+^); 129/Ola (129/Ola mPrP^+/+^) Transgenic mice: mPrP^0/0^ expressing hamster PrP^c^ transgene in neurons (NSE-HPrP/PrP^0/0^ mice); mPrP^0/0^ expressing hamster PrP^c^ transgene in astrocytes (GFAP HPrP/mPrP^0/0^ mice)	Surgery (for EEG and EMG electrode implant) Sleep deprivation EEG and EMG analysis Samples: blood (measurement of plasma corticosterone and adrenocorticotropic hormone levels)	The hormonal regulation of the hypothalamic–pituitary–adrenal axis, dependent on neuronal PrP^c^, may contribute to the maintenance of sleep homeostasis	[111]
	C57BL/6 mice (male, 3 months of age) (for sleep deprivation protocol) *Prnp* knock-out mice (used in neurite outgrowth assay)	Sleep deprivation Samples: hippocampus Western blot Immunoenzymatic assay (ELISA) Aβ peptides oligomerization (non-denaturing PAGE; size exclusion chromatography) Silver staining Binding assay Neurite outgrowth assay	Sleep deprivation influences the presence of PrP^c^ and Aβ peptides, potentially disrupting the interaction between PrP^c^ and laminin, as well as impairing neuronal plasticity	[112]
		Blood sample collection and processing; polygraphic tracings; measurement of plasma melatonin by RIA after diethyl ether extraction; analysis of rhythmicity	A gradual disturbance in the circadian rhythm of melatonin is noticeable in fatal familial insomnia	[114]
		Clinical data collection: clinical history, diagnostic data (CSF, EEG, MRI, laboratory values and PSG for a subset); CSF, EEG and MRI analyses; sleep data collection and analysis	Sleep abnormalities are prevalent in Creutzfeldt–Jakob disease, and it is recommended to include screening for sleep issues when assessing patients with rapidly progressing dementias	[115]
		Polysomnography recordings; PSG scoring	Sleep-related issues are a common and noteworthy aspect of the clinical presentation in both sporadic and familial forms of CJD.	[116]
	Rats (60–80 g body weight)	Inoculation with different strains of scrapie Electrode implantation (for EEG recording) EEG and EMG recording Sleep–wakefulness cycle classification (five states) Neuropathological examination	Rats inoculated with various prion strains demonstrate significant decreases in slow-wave sleep	[117]
	Rhesus monkeys	Electrode implantation (for EEG recording) Inoculation with a strain of Kuru EEG recording	Rhesus monkeys inoculated with a strain of Kuru exhibit a complete loss of REM sleep and disrupted sleep stage cycling	[118]
	HD line mice (mixed C57BL/6J and CBA genetic background) C57BL/6 mice (males)	Intracranial injection (with 30 μL of 0.1% uninfected mouse brain homogenate or 0.1% RML scrapie-infected mouse brain homogenate containing ≈ 5.5 log ID50 per 30 μL) Video recording (prion-infected mice were recorded for two consecutive 24 h periods at different time points post-inoculation)	Mice inoculated with the murine prion disease RML display alterations in rest period activity	[119]

## Data Availability

Not applicable.
